# *Helicobacter pylori* outer membrane vesicles and infected cell exosomes: new players in host immune modulation and pathogenesis

**DOI:** 10.3389/fimmu.2024.1512935

**Published:** 2024-12-13

**Authors:** Xiuping Wang, Jianjun Wang, Lingxiang Mao, Yongliang Yao

**Affiliations:** Department of Clinical Laboratory, The First People’s Hospital of Kunshan, Kunshan, Jiangsu, China

**Keywords:** *Helicobacter pylori*, exosome, outer membrane vesicles, immunomodulation, inflammation

## Abstract

Outer membrane vesicles (OMVs) and exosomes are essential mediators of host-pathogen interactions. Elucidating their mechanisms of action offers valuable insights into diagnosing and treating infectious diseases and cancers. However, the specific interactions of *Helicobacter pylori* (*H. pylori*) with host cells via OMVs and exosomes in modulating host immune responses have not been thoroughly investigated. This review explores how these vesicles elicit inflammatory and immunosuppressive responses in the host environment, facilitate pathogen invasion of host cells, and enable evasion of host defenses, thereby contributing to the progression of gastric diseases and extra-gastric diseases disseminated through the bloodstream. Furthermore, the review discusses the challenges and future directions for investigating OMVs and exosomes, underscoring their potential as therapeutic targets in *H. pylori*-associated diseases.

## 1 Introduction

*Helicobacter pylo*ri (*H. pylori*) infection is a prevalent chronic bacterial infection that colonizes the human gastric mucosa, leading to gastrointestinal disorders such as gastritis, peptic ulcers, and gastric cancer (1). As a highly adapted pathogen, *H. pylori* has evolved intricate mechanisms to modulate host immune responses and establish persistent colonization in the gastric environment (2). Recently, attention has shifted toward outer membrane vesicles (OMVs) released by *H. pylori* and exosomes derived from infected host cells, which play pivotal roles in host-pathogen interactions.

**Figure 1:**
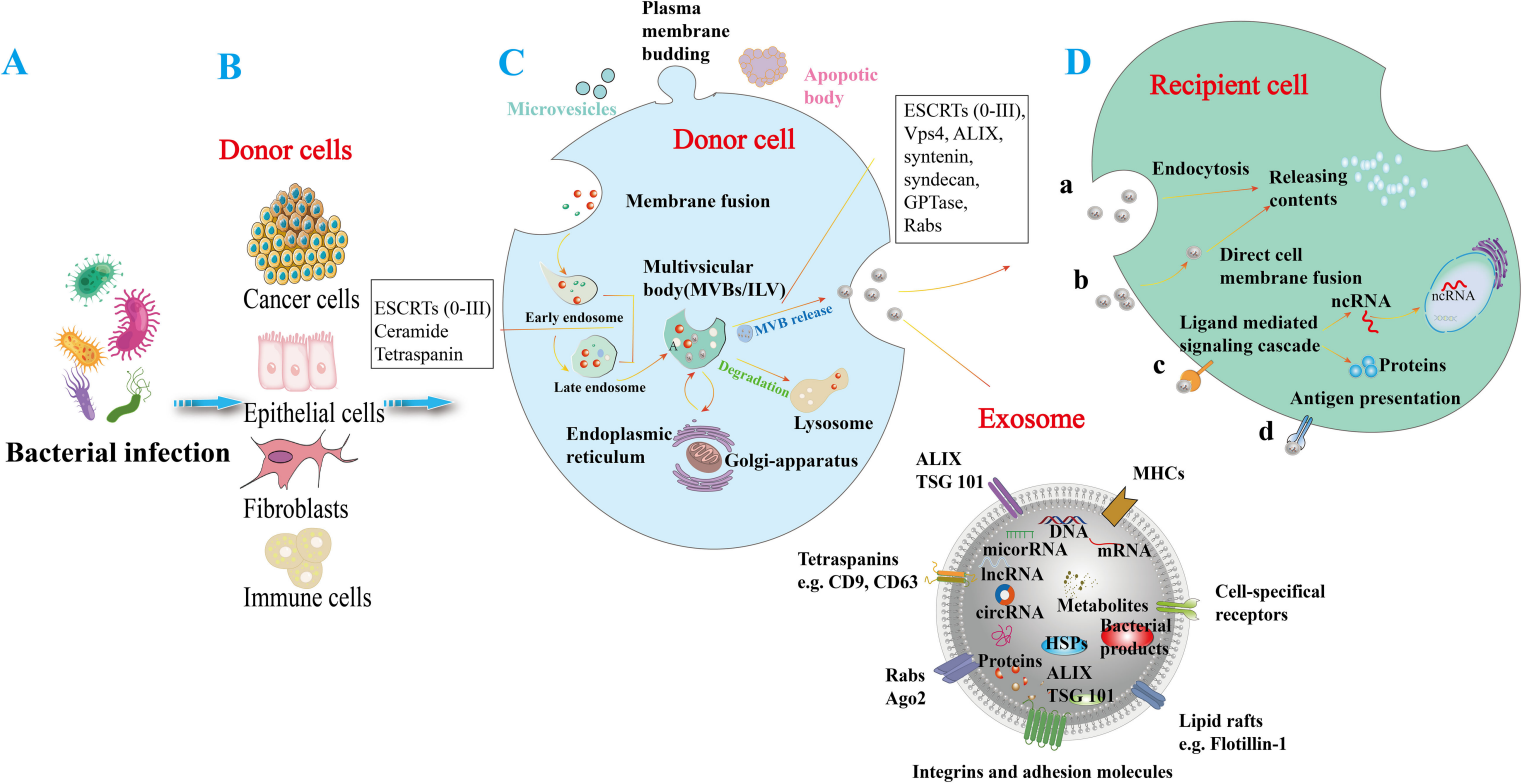
Generation and release of exosomes in response to bacterial infection. **(A)** Bacterial infections in various cell types can induce the release of exosomes. **(B)** Various bacterial-infected cell types, including cancer cells, epithelial cells, fibroblasts, and immune cells, can induce the release of exosomes. **(C)** Microvesicles arise from the outward budding of the plasma membrane, while apoptotic vesicles are lipid bilayer structures generated during programmed cell death. The process of exosome formation begins with the invagination of the plasma membrane to create early endosomes, which then mature into late endosomes. These endosomes produce intraluminal vesicles (ILVs) and utilize biogenesis proteins (e.g., ESCRTs, ALIX, syntaxin, and Rabs) to form multivesicular bodies (MVBs). MVBs can either merge with lysosomes for degradation or fuse with the cell membrane to release their enclosed vesicles into the extracellular space, resulting in exosome release. Exosomal cargo can include various molecules such as DNA, mRNA, small RNAs (miRNA, lncRNA, circRNA), proteins (e.g., heat shock proteins (HSP), ALIX/TSG101), metabolites, and bacterial components. Exosomes are composed of major histocompatibility complexes (MHC), specific cell receptors, lipid rafts, integrin adhesion molecules, Rabs, Ago proteins, tetraspanin proteins, and ESCRT proteins (e.g., ALIX/TSG101). These surface molecules may facilitate the uptake of exosomes by recipient cells. **(D)** Exosomes can exert their effects through various mechanisms, including endocytosis (a), direct fusion with the recipient cell membrane (b), ligand- and receptor-mediated signaling pathways (c), and antigen presentation (d), which release active molecules such as non-coding RNAs and proteins into target cells.

OMVs are nanoscale structures packed with virulence factors that modulate host immune responses and promote bacterial survival (3, 4). They facilitate the dissemination of bacterial antigens across the gastric epithelial barrier, influencing local and systemic inflammatory responses (3). Similarly, exosomes produced by *H. pylori*-infected epithelial cells carry molecular signatures reflective of the infection status and can alter immune cell signaling. In particular, exosomes derived from immune cells are crucial mediators of host-pathogen interactions and modulate immune responses significantly. Numerous studies have underscored the importance of these nanosized vesicles, demonstrating their ability to transport bioactive molecules, including proteins, lipids, nucleic acids, and metabolites (5). Research indicates their involvement not only in normal immune responses but also in modifying the tumor microenvironment and influencing the progression of autoimmune diseases (6). By delivering molecular cargo to recipient cells, exosomes are instrumental in the pathogenesis of various diseases, highlighting their critical function in immune system dynamics (7, 8) (**Figure 1**). Recent studies further emphasize exosomes’ role in mediating communication between *H. pylori* and host cells, significantly impacting disease progression (9, 10). Despite extensive research on OMVs and exosomes in various bacterial infections (3, 10–12), there is a significant gap in understanding their roles in *H. pylori* pathogenesis. This review aims to fill this gap by examining the unique interactions between *H. pylori*-derived OMVs, exosomes, and host immune responses, providing novel insights into their contributions to disease progression. Moreover, investigating the roles of OMVs and exosomes in *H. pylori*-related diseases, such as gastric cancer, offers opportunities for developing new diagnostic markers and therapeutic strategies.

This review focuses explicitly on the underexplored functions of *H. pylori*-derived OMVs and exosomes in host immune modulation, intending to stimulate further research and foster innovative management strategies for infections caused by this prevalent bacterium. Additionally, it elucidates how these vesicles contribute to the chronic nature of *H. pylori* infections and their potential implications for disease development.

## 2 Roles of *H. pylori* OMVs

### 2.1 Mechanisms of OMVs biogenesis

**Figure 2:**
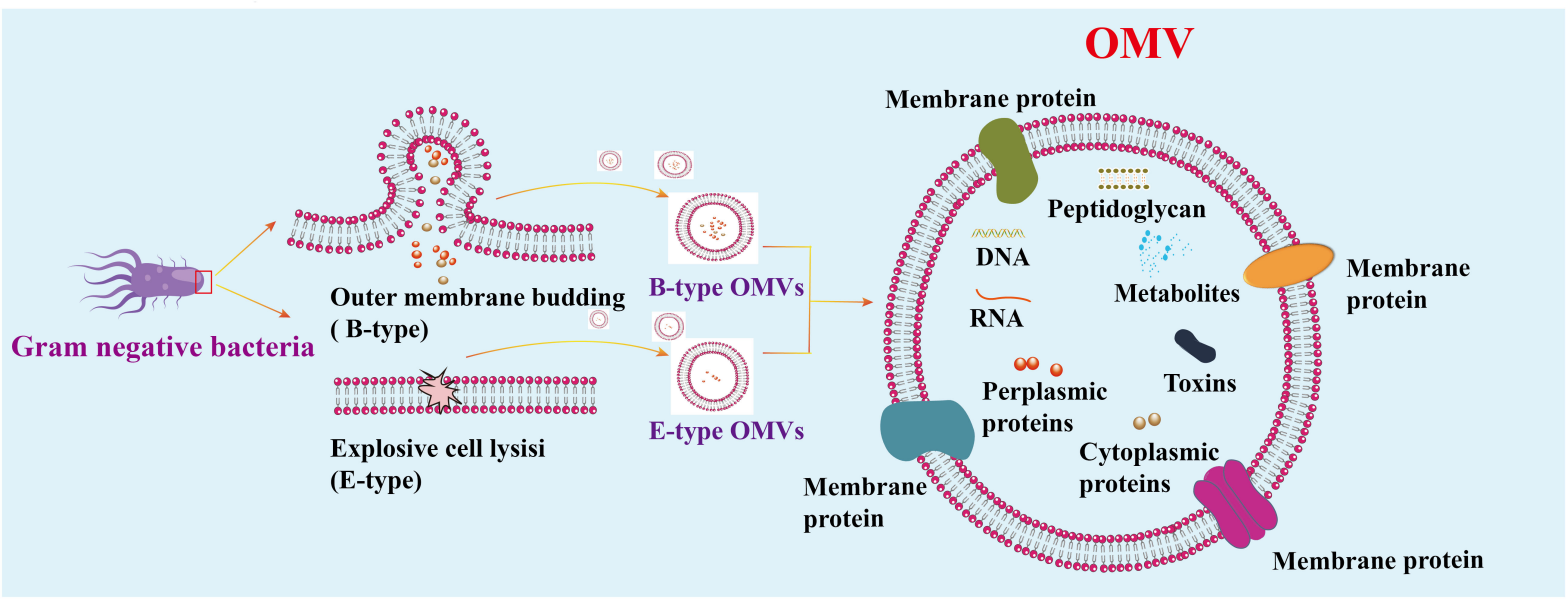
Mechanism of vesicle generation in Gram-negative. Gram-negative bacteria produce two types of OMVs: Type B and Type E. Type E OMVs are generated when explosive cell lysis disrupts the outer membrane. OMVs consist of a continuous lipid bilayer derived from the outer membrane and contain various components, including cytoplasmic and outer membrane proteins, toxins, enzymes, nucleic acids, peptidoglycans, and biomolecules from the parent bacterial cells.

During infection, pathogens directly release vesicles known as membrane vesicles. Gram-negative bacteria employ two main pathways to produce vesicles. The first pathway involves budding off the capsule membrane to form outer membrane vesicles (B-type OMVs). The second pathway requires explosive cell lysis to produce inner vesicles and explosion-derived outer membrane vesicles (E-type OMVs) (13, 14). The membrane composition of OMVs is generally similar to that of the bacterial outer membrane, although not always identical. Some outer membrane components are concentrated in OMVs, while others are absent or diminished. These vesicles, ranging in size from 20 to 500 nm, are crucial for bacterial communication with the environment and significantly contribute to bacterial pathogenesis (15–19) (**Figure 2**). Like other Gram-negative bacteria, *H. pylori* release OMVs from its outer membrane. The mechanisms regulating OMV production during *H. pylori* infections are currently under intense scrutiny. Understanding the precise pathways and regulatory processes involved in OMVs biogenesis during *H. pylori* infection is essential for grasping the implications of OMVs-mediated communication on disease progression.

### 2.2 Functional roles of *H. pylori* OMVs in host cells

*H. pylori* OMVs are nanoscale structures critical in bacterial pathogenesis (20). The size, composition, and protein cargo of OMVs vary with the growth stage of *H. pylori*, influencing their immunogenicity (17). Proteomic analyses have revealed numerous proteins in OMVs, including membrane proteins, porins, adhesins, and toxins, that facilitate interactions with host cells like those of intact bacteria (21). These OMVs elicit robust humoral and mucosal immune responses, predominantly biased toward Th2, making them potential vaccine candidates against *H. pylori* infection (22).

Moreover, *H. pylori* OMVs possess biologically active compounds that can be internalized by host cells, affecting signaling pathways and promoting apoptosis (3). For instance, they stimulate interleukin-8 (IL-8) secretion through NF-κB activation, contributing to inflammation (23). Additionally, *H. pylori* OMVs can enhance and downregulate immune responses, contributing to disease progression (3). Notably, *H. pylori* OMVs inhibit autophagy in hepatic stellate cells by altering the expression of autophagy-related genes, suggesting a novel mechanism for *H. pylori*-induced liver disorders (20). These findings underscore the complex involvement of *H. pylori* OMVs in modulating host cell functions and highlight their potential as therapeutic targets.

## 3 Roles of exosomes derived from *H. pylori*-infected cells

### 3.1 Mechanisms of exosomes formation

Exosomes are vesicles measuring 30-200 nm, encapsulated by a lipid bilayer with a density ranging from 1.13 to 1.19 g/mL (24, 25). Exosomes form through the endosomal pathway via the fusion of multivesicular bodies (MVBs) with the plasma membrane, resulting in the release of intraluminal vesicles (ILVs) (26). Lipids, proteins, and nucleic acids are transferred to the MVBs before entering vacuoles or being released as vesicles after fusion with the plasma membrane (24). Exosomes are integral to the endosomal sorting complexes required for transport (ESCRT) and may also form through an ESCRT-independent pathway to produce exosomes and microvesicles. GTP enzymes, along with proteins like Rabs, ESCRT complexes (ESCRT-0, I, II, and III), Syntenin-1, tumor susceptibility gene 101 (TSG101), programmed cell death 6 interacting (ALIX), syndecan-1, phospholipids, Tetraspanins (CD9 and CD63), ceramides, sphingosine, and soluble N-ethylmaleimide-sensitive factor (NSF) attachment protein receptor proteins (SNARE), play crucial roles in the biogenesis and release of exosomes (27, 28). The mechanisms underlying exosome formation and regulation during bacterial infections remain under investigation; however, it is recognized that exosomes carry specific cargo and can be utilized by bacteria (**Figure 1**).

### 3.2 Regulation of exosome secretion in *H. pylori*-infected cells

Exosomes are also considered novel diagnostic biomarkers in various pathological contexts, including bacterial infections (29). *H. pylori* is known to regulate exosome secretion from host cells to establish chronic infection (30). Understanding how *H. pylori* facilitates or impedes exosome release from host cells is essential for deciphering the pathogenesis of *H. pylori*-associated diseases. *H. pylori* infection increases exosome release, potentially activating various signaling pathways through virulence factors such as cytotoxin-associated gene A (CagA) and vacuolating cytotoxin A (VacA) (10, 11, 30). CagA induces pro-inflammatory cytokines and chemokines expression, potentially enhancing the secretion of inflammatory exosomes (12). VacA alters the composition and function of host-derived exosomes, which may assist bacterial survival and colonization in the gastric mucosa (30–32). *H. pylori* infection triggers various cellular stress responses, including endoplasmic reticulum and oxidative stress, associated with increased exosome secretion (30). These stress signals may prompt exosome release as a cellular communication mode and response to the pathogen. Conversely, *H. pylori* may disrupt normal cellular signaling pathways associated with exosome biogenesis and release, inhibiting exosome secretion (30). Furthermore, *H. pylori* impacts the lipid composition of host cell membranes, thereby affecting exosome formation and release (30). Alterations in lipid composition may obstruct exosome budding and release, thereby hindering secretion. The precise mechanisms by which *H. pylori* promotes or inhibits exosome release from host cells are still being elucidated and may vary based on the bacterial strain and host cell type. Future research exploring the regulation of exosome secretion by *H. pylori* virulence factors may entail examining the specific signaling pathways and molecular interactions involved in this process. Additionally, understanding the content of *H. pylori*-modulated exosomes and their impact on host immune cells and epithelial cells is crucial for unraveling the intricate mechanisms of bacterial manipulation of host exosomal machinery.

### 3.3 Functional roles of exosomes in *H. pylori* infection

*H. pylori* utilizes exosomes to transport virulence factors, genetic material, and bioactive molecules to host cells, primarily targeting gastric epithelial cells and immune cells, including macrophages and dendritic cells (30, 33, 34). This specificity is vital for the bacterium’s ability to modulate the host immune response, facilitating its survival and persistence (11, 34, 35). Exosomes derived from *H. pylori*-infected cells deliver key virulence factors, such as CagA and VacA, which directly influence host cell signaling pathways, altering cellular functions and immune responses (36). This delivery can induce significant changes in cell proliferation, apoptosis, and immune activation (30). Transferring bacterial DNA and RNA via exosomes can instigate genetic and epigenetic modifications in recipient cells, ultimately shaping the host’s response to *H. pylori* infection (37, 38).

An intriguing aspect of exosome biology in the context of *H. pylori* infection is their potential role in mediating the bacterium’s toxicity. Exosomes derived from *H. pylori*-infected cells contribute significantly to antibiotic resistance development and the establishment of a pro-inflammatory environment in the gastric mucosa (10, 11, 39), triggering the release of pro-inflammatory cytokines and chemokines that contribute to chronic inflammation, tissue damage, gastritis, and progression to severe *H. pylori*-related diseases (11, 31). These exosomes could act as shuttles for delivering bacterial toxins or effectors to adjacent cells, enhancing *H. pylori*’s overall virulence and exacerbating tissue damage. They also have the potential to be used as drug-delivery vehicles to improve treatment outcomes (40). To target *H. pylori*-infected cells, a proposed approach involves using exosomes derived from macrophages capable of recognizing infected cells, eradicating pathogenic microorganisms, activating innate immune responses, and delivering drugs against *H. pylori* (40). This method effectively enhances treatment efficiency while sparing healthy stomach cells from side effects. In cancer, exosomes facilitate intercellular communication within the tumor microenvironment (TME) and contribute to drug resistance by transferring specific mRNA, ncRNA, or proteins, presenting therapeutic targets (41, 42). Moreover, transferring genetic material, including antibiotic-resistance genes, among bacterial populations through exosomes could potentially spread resistance, posing challenges to antibiotic therapy for *H. pylori* and its evolving resistance issue (43). Understanding the interplay between exosomes and *H. pylori* in the context of drug resistance is crucial to developing novel therapeutic strategies against this persistent pathogen.

### 3.4 Crosstalk between *H. pylori* and host cells mediated by exosomes

Transiting *H. pylori*’s pathogenic signals via exosomal cargo offers insights into how the bacterium manipulates host cell responses to establish persistent infection and evade immune surveillance (11, 35). Additionally, *H. pylori* infection may alter the composition of exosomes originating from infected cells, influencing the microenvironment and regulating immune responses (44). *H. pylori-*infected cell exosomes can modulate host cell gene expression profiles by delivering bioactive molecules such as microRNAs (miRNAs) and other regulatory factors, which interact with specific receptors and signaling pathways, triggering downstream cellular responses that promote inflammation, alter gene expression, and induce host cell damage (37). The delivery of these molecules can affect different cellular processes, such as inflammation, cell proliferation, and immune responses, resulting in host cell function dysregulation and the advancement of gastric conditions like chronic gastritis and gastric cancer (37, 45, 46). The involvement of miRNAs in *H. pylori*-triggered infections and gastric cancers is continuously under investigation, holding promise for the advancement of early preneoplastic stages (47). Aberrant miRNAs in *H. pylori*-related inflammation and carcinogenesis could be non-invasive biomarkers (48). These findings underscore the ability of *H. pylori* to reprogram host cellular responses and establish a microenvironment conducive to bacterial survival and disease progression (34).

## 4 The interaction between *H. pylori* OMVs and exosomes

Recent literature underscores the critical role of *H. pylori* OMVs in pathogenesis and elucidates the function of exosomes in intercellular signaling, particularly how bacterial virulence factors can influence exosome content. Infected cell exosomes and OMVs do not always contain bacterial components, but they can carry bacterial materials and host-derived molecules, including post-translationally modified proteins. *H. pylori*-released OMVs are rich in bioactive molecules, including proteins, lipids, and nucleic acids, directly engaging with host cells. This unique composition enables OMVs to provoke inflammatory responses and facilitate *H. pylori* invasion of host tissues (3, 10). Notably, virulence factors, such as VacA and CagA, found within OMVs, play critical roles in immune evasion and the induction of apoptosis in host cells (3). Furthermore, the lipid constituents of OMVs can alter the physical properties of host cell membranes, promoting vesicle fusion with target cells and the subsequent delivery of bioactive cargo (49). Conversely, exosomes are pivotal in mediating intercellular communication and orchestrating immune responses. Exosomes secreted by *H. pylori*-infected cells transport host-derived proteins and microRNAs that can modulate immune activity, suppressing or promoting host defenses against the infection (10). Exosome-derived microRNAs specifically target and regulate the expression of key mRNAs in host cells, resulting in significant alterations to the immune response landscape. **Table 1** compares the major components of the most studied molecules and proteins in OMVs and exosomes.

**Table 1:** Comparison of the major components and pathological implications of OMVs and exosomes in *H. pylori* infection.

Component	OMVs	Exosomes	Interaction and pathological implications in *H. pylori* infection	References
Size	Typically 20-500 nm	Typically 30-200 nm	Size influences uptake by host cells	(15–19, 24, 25)
Origin	Derived from the outer membrane of bacteria	Derived from the endosomal pathway of host cells	Reflects different pathways of immune modulation	(13, 14)
Key proteins	Contains virulence factors (e.g., VacA, CagA)	Contains host-derived proteins and microRNAs	Key roles in immune evasion and modulation	(5, 50, 51)
Lipids	Rich in phospholipids and lipopolysaccharides	Rich in cholesterol and sphingomyelin	Lipid composition affects membrane stability and interactions	(5, 52)
Nucleic acids	Minimal RNA content	Contains various microRNAs	MicroRNAs in exosomes play roles in regulating host immune responses	(5)
Main functions	Elicit inflammatory responses, facilitate invasion	Mediate communication, modulate immune responses	Influence the immune landscape during infection	(3–6, 24)
Immune modulation	Induces apoptosis and immune evasion	Alters expression of mRNAs in target cells	Both contribute to *H. pylori*’s ability to evade host defenses	(3–6, 24)
Pathological implications	Promotes disease progression in gastric and extra-gastric conditions	Implicated in fibrosis and liver disease	Reflects systemic impact on host physiology	(20, 53–55)

Evidence suggests that modifications in exosomal cargo during bacterial infections represent a novel mechanism of systemic pathogenesis (53, 54). For instance, exosomes from *H. pylori* OMV-infected hepatocytes have been shown to induce the expression of α-smooth muscle actin (α-SMA), tissue inhibitor of metalloproteinases-1 (TIMP-1), β-catenin, and vimentin in hepatic stellate cells (HSCs), while concurrently downregulating E-cadherin gene and protein levels (55). These findings indicate that *H. pylori* OMVs may affect exosome composition, thereby contributing to HSC activation and the progression of liver fibrosis. Exploring the intricate mechanisms of interaction between *H. pylori* OMVs and exosomes holds promise for unveiling novel therapeutic strategies for *H. pylori*-associated diseases. However, the mechanisms underlying these interactions remain unclear due to insufficient research.

## 5 Modulation of *H. pylori* OMVs and infected cell exosomes on immune responses

**Figure 3:**
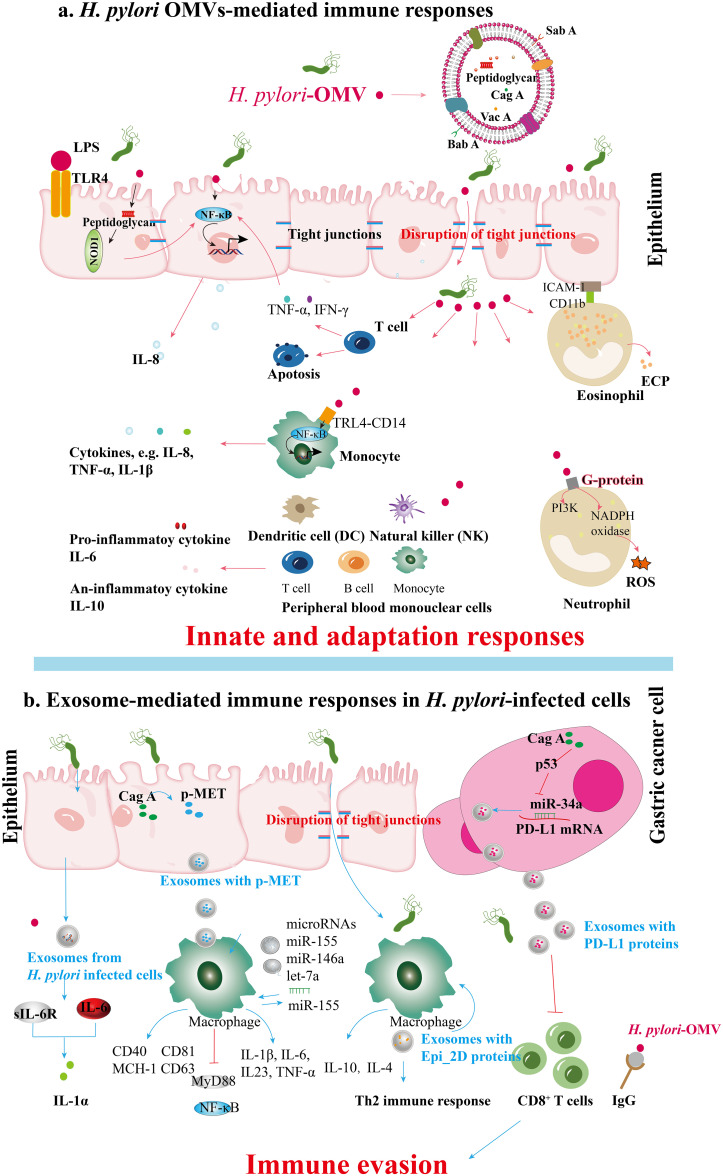
Immune responses mediated by (*H*) *pylori* OMVs and infected cell exosomes. **(A)** BabA and SabA, found in *H*. *pylori* OMVs, enhance adhesion to the gastric mucosa. Peptidoglycan within *H*. *pylori* OMVs activates the NOD1 response. During *H*. *pylori* infection, OMVs induce the secretion of cytokines like TNF-α and IFN-γ from T cells and stimulate gastric epithelial cytokine release (e.g., IL-8) via NF-κB activation. OMV ligands binding TLR4 and CD14 on monocytes activate NF-κB, enhancing cytokine release. *H*. *pylori* OMVs upregulate ICAM-1 and CD11b integrin expression, leading to degranulation and ECP release; these OMVs also activate NADPH oxidase in neutrophils through GPCR signaling and kinase pathways, inducing oxidative burst and ROS production. Furthermore, *H*. *pylori* OMVs can induce serum IgG responses, potentially aiding immune evasion during *H*. *pylori* pathogenesis. **(B)** Exosomes from *H*. *pylori-*infected human gastric epithelial cells secrete GES-1, inducing the expression of soluble IL-6 receptor that mediates the promotion of proinflammatory cytokines such as IL-1α. CagA promotes the disruption of intercellular tight junctions. After *H*. *pylori* infection, vesicles containing p-MET are released. Macrophages subsequently take up these vesicles, which release proinflammatory cytokines that enhance tumor growth and stimulate cell proliferation, migration, and invasion. Apart from the cell signaling proteins CD81, CD63, CD40, and MCH-I, exosomes containing miR-155 from *H*. *pylori*-infected macrophages also enhance the secretion of inflammatory cytokines IL-23, IL-6, IL-1β, and TNF-α. Simultaneously, miR-155 expression activates MyD88 signaling pathways and NF-κB inflammatory protein in macrophages during *H*. *pylori* infection. The exosomes derived from *H*. *pylori-*infected cells contain a specific protein, Epi_2D, which triggers a Th2 immune response, producing IL-4 and IL-10. *H*. *pylori*’s CagA inhibits p53 and miR-34a, leading to the upregulation of PD-L1 levels in gastric cancer cells, which inhibits CD8^+^ T cell proliferation and anti-cancer response, promoting immune evasion in gastric cancer.

Understanding the specific components of OMVs and exosomes that contribute to *H. pylori* pathogenesis is crucial for elucidating their roles in inducing immune suppression and the persistence of infection in the host immune environment (**Table 2**). This review provides an in-depth look at how OMVs released by infected gastric epithelial cells can mobilize and stimulate immune cells, including macrophages and T cells, fostering the persistent inflammatory environment typical of *H. pylori*-related conditions. Conversely, exosomes originating from *H. pylori*-infected cells can alter the activity of immune cells, fostering an immunosuppressive setting conducive to bacterial colonization and the circumvention of host immune monitoring (**Figure 3**).

**Table 2:** Key components of *H. pylori* OMVs and infected cell exosomes involved in pathogenesis and immune response.

Component	Type	Role in pathogenesis /immune response	Mechanistic Insights	Significance	References
VacA	OMV	Induces vacuole formation, modulates immune response, and promotes apoptosis in host cells	VacA enters host cells through endocytosis, resulting in the formation of large vacuoles. It inhibits T-cell activation and induces apoptosis in various immune cells, thereby contributing to immune evasion	Facilitates immune evasion and contributes to gastric pathology	(50, 56–59)
CagA	OMV/ Exosome	Alters host cell signaling pathways promote inflammation and are associated with gastric cancer	Disrupts host cell signaling and enhances inflammation	Enhances bacterial virulence and contributes to disease progression	(30, 51)
GGT	OMV	Induces immune tolerance	Contributes to cell-cycle arrest, apoptosis, and necrosis in gastric epithelial cells. Through the inhibition of T cell-mediated immunity and dendritic cell differentiation	Favoring persistent infection and gastric colonization	(34, 60–63)
LPS	OMV	Triggers strong inflammatory responses in host immune cells	Key roles in immune evasion and modulation	Plays a role in the inflammatory response to *H. pylori* infection	(5, 52, 64)
Outer membrane proteins (OMPs)	OMV	Involved in adhesion to host cells and immune evasion mechanisms	Facilitate host cell adhesion, immune evasion, and modulation of inflammatory responses	Critical for establishing and maintaining infection	(21, 65)
Exosomal microRNAs	Exosome	Modulate host immune responses and can induce immunosuppressive environments	Exosomal microRNAs derived from *H. pylori* can modulate the expression of genes involved in immune regulation and inflammation, enhancing bacterial persistence	Influence gene expression in host cells, contributing to immune modulation	(9, 37, 48, 66)
Phosphorylated mesenchymal-epithelial factor (p-MET)	Exosome	contributing to epithelial cell transition and inflammatory responses	Macrophages internalize this factor, resulting in elevated levels of IL-1β and IL-6 mRNA and increased IL-1α secretion, thereby fostering tumor growth	The presence of p-MET highlights the role of *H. pylori* in modulating host cell signaling pathways	(12)

### 5.1 Immune cell activation and immune tolerance modulation by *H. pylori* OMVs and infected cell exosomes

OMVs derived from Gram-negative bacteria, including *H. pylori*, carry various pathogen-associated molecular patterns (PAMPs), such as lipopolysaccharides (LPS), lipoproteins, peptidoglycans, and bacterial nucleic acids. These PAMPs interact with pattern recognition receptors (PRRs) on host immune cells, particularly epithelial cells in mucosal tissues, influencing host pathology, immune tolerance, and protective immunity (64). *H. pylori* OMVs activate Toll-like receptor (TLR) signaling pathways, resulting in the release of pro-inflammatory cytokines such as interleukin-1β (IL-1β), tumor necrosis factor-alpha (TNF-α), and interleukin-6 (IL-6), which attract immune cells to the infection site (67, 68). This cytokine release is critical in mediating mucosal damage caused by *H. pylori*, as it triggers the secretion of chemokines and the activation of nuclear factor-kappa B (NF-κB) (69). Components of *H. pylori*, including LPS, heat shock protein 60 (HSP60), and nucleic acids, are recognized via various TLRs (70). The subsequent activation of NF-κB and mitogen-activated protein kinase (MAPK) pathways plays a key role in regulating pro- and anti-inflammatory responses, contributing to the immune response and disease progression (70). Cytoplasmic receptors such as nucleotide-binding oligomerization domain 1 (NOD1) and NOD2 recognize OMVs containing peptidoglycan, a major bacterial cell wall component, thus initiating critical innate immune responses (71). Activating NF-κB by *H. pylori* OMVs that engage NOD1 can increase IL-8 expression, potentially contributing to various gastric disorders (23). During T-cell activation, *H. pylori* infection promotes the secretion of TNF-α and interferon-gamma (IFN-γ) through NF-κB signaling, which stimulates gastric epithelial cells to produce IL-8 (72, 73). The binding of *H. pylori* OMVs to TLR4 and CD14 on mononuclear cells triggers NF-κB activation and subsequent cytokine release. Moreover, components within *H. pylori* OMVs can induce both pro-inflammatory cytokines (e.g., IL-6) and anti-inflammatory cytokines (e.g., IL-10), while also initiating oxidative bursts and apoptosis in Jurkat T cells (74, 75). This oxidative burst is mediated by the activation of nicotinamide adenine dinucleotide phosphate (NADPH) oxidase through components like neutrophil-activating protein A (NAP), leading to upregulation of β2 integrin expression and attraction of leukocytes to epithelial cells (76, 77). Ultimately, this process induces degranulation in eosinophils, resulting in the release of eosinophil cationic protein (ECP) and increased expression of ICAM-1 on epithelial cells and CD11b integrin on eosinophils (78) (**Figure 3A**).

Exosomes released from infected cells, including those during pathogen infections, have been shown to contain microbial components that modulate host immunity (24, 79). These exosomes can enhance antigen presentation and stimulate macrophages, potentially influencing disease progression (80). In the case of *H. pylori*, the bacterium can manipulate the release and content of exosomes to promote its survival and colonization in the gastric mucosa, indicating a complex mechanism of exosome-mediated communication that supports chronic infection. Che et al. (2018) investigated the interactions facilitated by exosomes between *H. pylori*-infected gastric cancer cells and macrophages. Their findings revealed that gastric cancer cells release phosphorylated mesenchymal-epithelial factor (p-MET) following *H. pylori* infection. Macrophages internalize p-MET, leading to increased levels of IL-1β and IL-6 mRNA and elevated IL-1α secretion, which fosters tumor growth (12). Another study focused on serum exosomes in the IL-6-mediated regulation of inflammatory cytokines. Analysis of exosomes from individuals with chronic gastritis infected with *H. pylori* showed upregulation of IL-1α and soluble IL-6 receptor (sIL-6R) in human gastric epithelial cells (GES-1). The pro-inflammatory role of IL-6, mediated by sIL-6R, promotes the secretion of additional inflammatory cytokines, while IL-1α contributes to the inflammatory response in various diseases (39, 81–83). Despite the limited number of studies, exosomes play a crucial role in the abnormal inflammatory response induced by *H. pylori* infection, with specific mechanisms still poorly understood. Exosomes from *H. pylori*-infected cells can modulate immune cell activation through various pathways, including the regulation by microRNAs such as miR-155, which influences the host immune response (9, 48). Research by Wang et al. (2016) and Atrisco-Morales et al. (2022) highlighted the role of exosomes in promoting inflammatory responses, particularly emphasizing miR-155’s involvement (9, 66). Furthermore, Chen et al. (2018) underscored the immunomodulatory functions of exosomes in educating tumor-associated macrophages (39). Recent studies by Faass et al. (2023) and Ahmed et al. (2021) explored the interactions between *H. pylori* and immune cells, with Faass et al. examining myeloid cell activation by *H. pylori* metabolites and Ahmed et al. identifying Epimerase_2 domain-containing protein (Epi_2D) in *H. pylori*-infected cell exosomes that induce a Th2 immune response (84, 85). These studies highlight the significant impact of exosomes derived from *H. pylori*-infected cells on immune cell activation (**Figure 3B**).

*H. pylori*-infected cell exosomes have been implicated in promoting the differentiation of T helper 17 (Th17) cells, known for producing pro-inflammatory cytokines like IL-17 (86). These exosomes can also shift immune cells toward a regulatory phenotype, inhibiting effector T-cell responses and fostering immune tolerance in the gastric mucosa (87). The immunological functions of exosomes, particularly their involvement in adjusting antigen presentation and immune activation, have been extensively studied (88). Moreover, *H. pylori* infection triggers a combined Th17/Th1 cell response, where the Th17/IL-17 pathway influences Th1 cell responses, contributing to disease progression (89). The role of regulatory T cells in responding to *H. pylori* infection has also been investigated, focusing on their role in the anti-bacterial immune response (90). Additionally, *H. pylori* infection-deriving exosomal proteins, including HSPs and virulence factors, can modulate host cell immune signaling pathways, fostering immune tolerance and persistent infection (91). These findings indicate that *H. pylori* OMVs and infected cell exosomes can impact the host’s immune response and play a role in chronic infection and immune tolerance.

### 5.2 Immune evasion modulation by *H. pylori* OMVs and infected cell exosomes

*H. pylori* OMVs play a significant role in immune evasion by inducing serum immunoglobulin G (IgG) responses, notably through antibodies that target bacterial Lewis epitopes (92). In hosts with cross-reactive blood group structures, these antibodies may contribute to the autoimmune processes associated with *H. pylori* pathogenesis. The differential packaging of host mRNA within OMVs as small non-coding RNAs (sncRNAs) further modulates the host immune response (93). For example, Zhang et al. found that sncRNA-2509025 and sncRNA-989262 could reduce IL-8 secretion triggered by lipopolysaccharide or OMVs in AGS cells cultured *in vitro*, thereby facilitating immune evasion (94). Additionally, Li et al. demonstrated that these sncRNAs play a crucial role in regulating the immune response in *H. pylori*-infected animal hosts by increasing serum IgG and IgA levels. Their depletion negatively impacted mucosal and humoral immunity, underscoring the direct role of sncRNAs in modulating host immune responses and aiding *H. pylori* in evading host defenses (95). These results suggest that sncRNAs within *H. pylori* OMVs directly modulate the host immune response, aiding *H. pylori* in evading host immunity. Nonetheless, additional investigations on host-pathogen interactions are necessary.

*H. pylori* employs various strategies to evade host immunity, including modifying surface molecules to avoid detection by innate immune receptors and modulating effector T-cell responses (2, 96, 97). Exosomes released during *H. pylori* infection serves a dual role, acting as an agent of immunosuppression and immune evasion (24). These exosomes deliver virulence factors and immunomodulatory molecules to host cells, subverting immune defenses and fostering an environment conducive to bacterial survival (98, 99). They promote the expansion of regulatory T cells and inhibit antigen presentation, creating a tolerogenic setting that facilitates bacterial colonization (98). Research has indicated a significant correlation between the presence of CagA protein in tumor tissues, high PD-L1 expression rates, elevated plasma PD-L1 levels, and lymph node metastasis in gastric cancer patients with *H. pylori* infection. Mechanistically, CagA hinders CD8^+^ T cell proliferation and anti-cancer responses by enhancing PD-L1 levels in exosomes from gastric cancer cells through p53 and miRNA-34a inhibition. Targeting CagA and extracellularly secreted PD-L1 could improve the efficacy of immunotherapy against *H. pylori*-related gastric cancer (51) (**Figure 3B**).

## 6 Role of *H. pylori* OMVs and infected cell exosomes in chronic inflammation and pathogenesis

The presence of confirmed and potential virulence factors in *H. pylori* OMVs suggests that these vesicles play a crucial role in delivering toxins and virulence factors to the gastric epithelium, contributing to the chronic inflammation associated with *H. pylori* infection. For instance, studies have shown that VacA associated with OMVs can induce cavitation in gastric epithelial cells (58, 59). Additionally, OMVs contain the virulence factor NAPA, which exerts a chemotactic effect on human neutrophils and monocytes, stimulating the production of reactive oxygen intermediates (ROIs) that can damage gastric epithelial cells and enhance nutrient release for the bacteria (76). The uptake of OMVs containing VacA increases cytoplasmic iron levels while concurrently reducing glutathione (GSH), creating an oxidative environment that elevates ROI levels and is associated with DNA damage. Internalized OMVs can induce apoptosis in gastric epithelial cells via a caspase-dependent, mitochondria-independent pathway, leading to decreased cell viability and DNA fragmentation, with activation of caspase-8, -9, and -3 observed despite the absence of cytochrome C release (57). Another virulence factor, gamma-glutamyl transpeptidase (GGT), induces cell cycle arrest, apoptosis, and necrosis by depleting GSH and generating reactive oxygen species, further promoting apoptosis in epithelial cells (61, 100). GGT also enhances *H. pylori* persistence and colonization by promoting immune tolerance by inhibiting T cell-mediated immunity and dendritic cell differentiation (62, 63).

**Figure 4:**
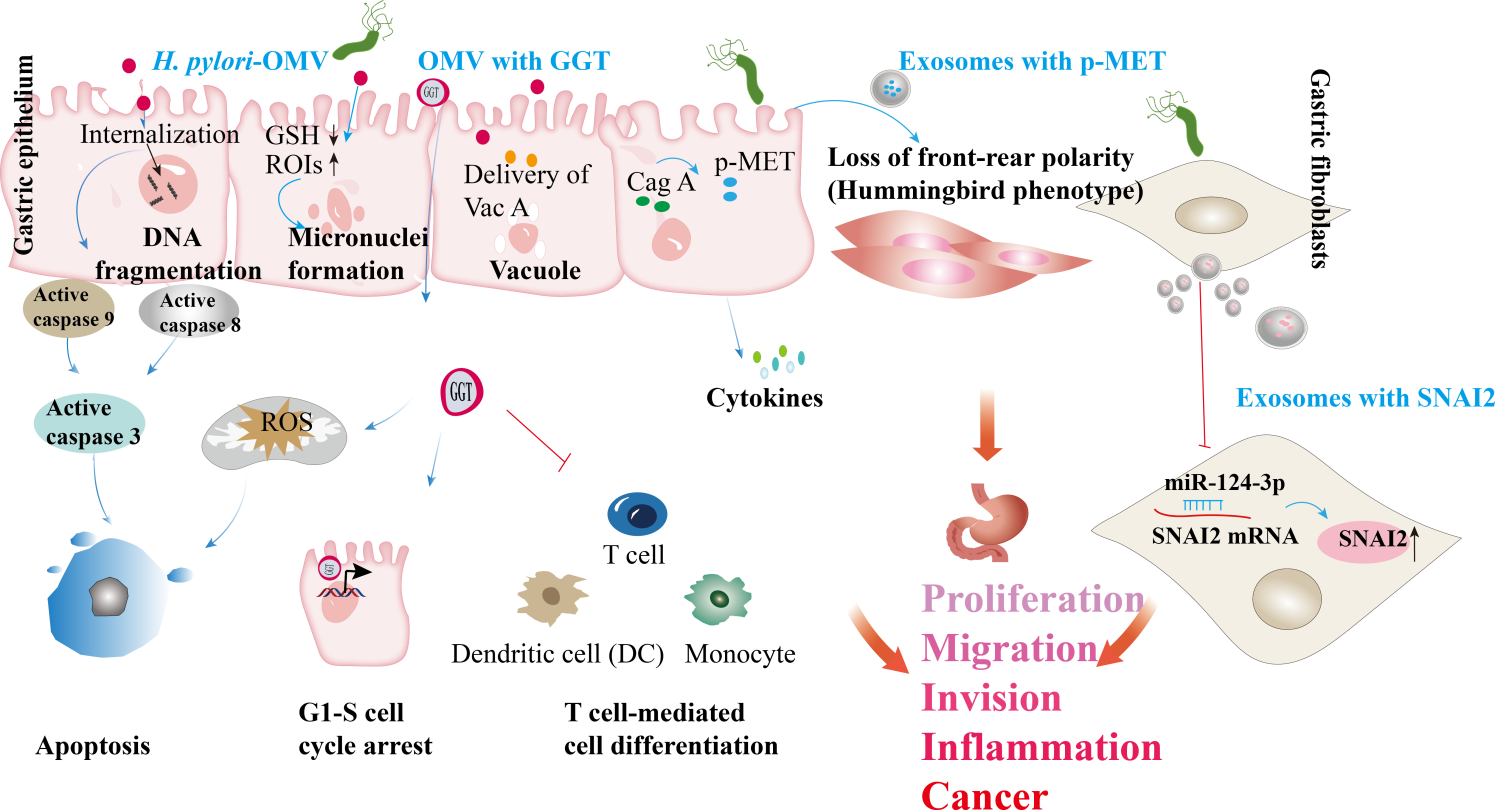
*H. pylori* OMVs and infected cell exosomes play a role in the development of gastric diseases. Internalizing *H. pylori* OMVs into epithelial cells triggers DNA fragmentation and activates caspase-8, 9, and 3. The absorption of OMVs carrying the VacA leads to reduced GSH levels (GSH↓). The absorption of OMVs carrying the VacA leads to reduced GSH levels. In this oxidative environment, reactive oxygen intermediates (ROIs) increase  (ROIs↑), contributing to DNA damage. GGT from *H. pylori* triggers the production of ROS, leading to cell apoptosis. GGT can induce cell cycle arrest via nuclear reactions and immune tolerance, suppressing T cell-mediated immunity and dendritic cell differentiation. *H. pylori* OMVs trigger the formation of micronuclei and vacuoles through VacA. *H. pylori* infecting host cell-derived exosomes has been linked to gastric cancer development. In *H. pylori* infection, the virulence factor CagA is delivered to gastric epithelial cells; released exosomes carrying phosphorylated CagA (p-CagA) trigger cellular morphology changes. Post *H. pylori* infection, the mesenchymal epithelium releases exosomes with p-MET, internalized by macrophages, triggering proinflammatory cytokine release and supporting tumor progression. *H. pylori* infection enhances the proliferation and migration of gastric fibroblasts (GFs) by promoting the secretion of exosomes containing miR-124-3p, which is associated with the SNAI2 protein levels (↑ indicates increased SNAI2 levels).

Exosomes released from H. *pylori*-infected cells can also induce the release of pro-inflammatory cytokines and chemokines, leading to tissue damage, gastritis, and progression to more severe *H. pylori*-related diseases (11, 31). For instance, these exosomes modulate the expression of IL-1α in gastric epithelial cells, contributing to chronic gastritis (12). In patients with *H. pylori* infection, CagA was isolated from serum samples, and its overexpression in gastric epithelial cells resulted in the release of exosomes containing phosphorylated CagA. Treatment of *WT*-A10 gastric epithelial cells with these exosomes induced an elongated cell morphology characteristic of the hummingbird phenotype (30). A different study revealed that gastric fibroblasts (GF) are direct targets of *H. pylori* (101). Moreover, gastric fibroblasts (GFs) are direct targets of *H. pylori*. *H. pylori*-activated GFs release exosomes with significantly increased levels of miR-124-3p, promoting the expression of cancer-associated fibroblast (CAF) biomarkers. *H. pylori* infection enhances GF proliferation and migration by facilitating the release of exosomes loaded with miR-124-3p, an inhibitor of SNAI2 (101). This exosomal communication influences the gastric microenvironment, fostering a pro-inflammatory and tumorigenic milieu that facilitates disease progression (11). Furthermore, exosomes facilitate the transfer of molecules such as activated MET protein, instructing tumor-associated macrophages to enhance gastric cancer progression (12). Exosomes from gastric cancer cells also contribute to forming pre-metastatic niches (102), while those from infected gastric epithelial cells can induce carcinogenic changes in recipient cells (103). Overall, these findings underscore the essential role of OMVs and exosome-mediated signaling in the pathogenesis of *H. pylori*-related gastric diseases (**Figure 4**).

**Figure 5:**
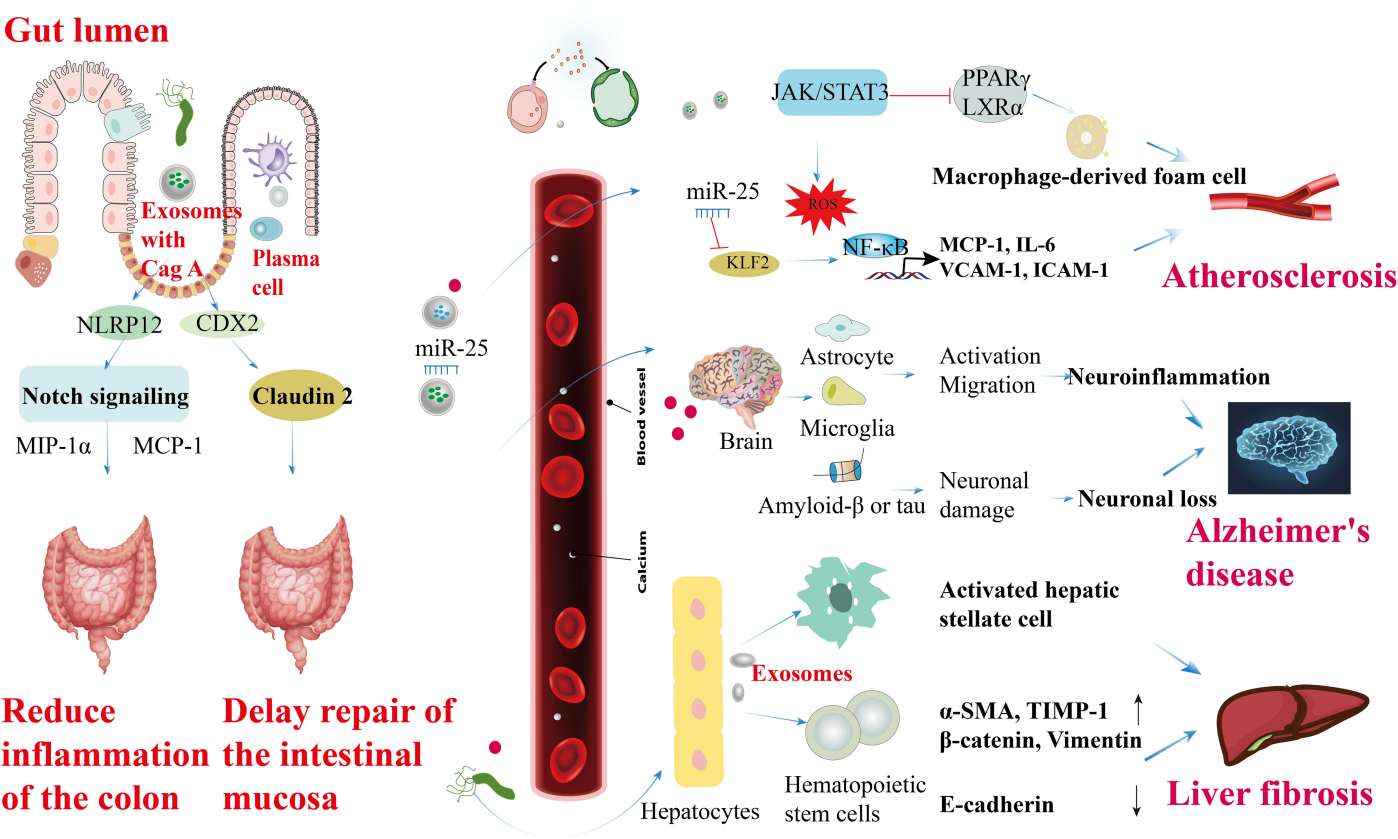
Roles of *H. pylori* OMVs and infected cell exosomes in the extra-gastric diseases. CagA-containing exosomes enhance NLRP12 expression in intestinal epithelial cells, inhibiting the Notch signaling pathway and reducing the levels of chemokines like MIP-1α and MCP-1, ultimately improving colitis symptoms. Extracellular secretion of CagA by CDX2-dependent mechanism from GES-1 modulates transcriptionally, correlating with claudin-2 protein expression to facilitate intestinal mucosal repair delays. CagA secretion from *H. pylori* in vascular endothelial cells induces ROS formation, leading to endothelial cell activation, JAK-STAT3 activation factor signaling disruption, promoting macrophage foam cell formation, and atherosclerosis development. Circulating miR-25-containing exosomes can modulate the NF-κB signaling pathway by targeting the transcription factor KLF2, subsequently increasing the expression of ICAM-1, MCP-1, VCAM-1, and IL-6, thus accelerating atherosclerosis in vascular endothelial cells. Furthermore, OMVs produced during chronic *H. pylori* infection in the intestinal tract activate microglia and astrocytes to promote their migration. This activation, along with the enhancement of amyloid β or tau, can trigger inflammation in the central nervous system, potentially elucidating the higher prevalence of Alzheimer’s disease in individuals with *H. pylori* infection. Lastly, *H. pylori* infection induces the expression of α-SMA, TIMP-1, β-catenin, and vimentin (α-SMA, TIMP-1, β-catenin, and vimentin ↑) in hematopoietic stem cells derived from hepatocytes, while downregulating the expression of E-cadherin at both the gene and protein levels (E-cadherin ↓), ultimately contributing to liver fibrosis.

*H. pylori* OMVs and exosomes released from infected cells can enter the bloodstream and reach other tissues, potentially causing extra-gastric ailments. Chronic stomach colonization by *H. pylori* produces OMVs that disrupt tight junctions in the intestinal epithelium, resulting in increased blood-brain barrier (BBB) permeability. This compromised BBB facilitates the infiltration of OMVs into the brain parenchyma, where they stimulate microglia and astrocytes, exacerbating neuronal damage associated with amyloid β or tau pathology and accelerating neurodegeneration around amyloid plaques, thereby contributing to Alzheimer’s disease (104). Exosomes serve as important carriers of *H. pylori* components, elucidating the mechanisms of how *H. pylori* induces localized and systemic changes. For instance, exosomes derived from plasma and gastric epithelial cells can transport *H. pylori* CagA into circulation, potentially promoting the progression of extra-gastric diseases (30). In the intestinal epithelium, exosomes from *H. pylori*-positive individuals increase levels of NOD-like receptor family pyrin domain containing 12 (NLRP12), which suppresses the expression of chemokines, such as monocyte chemoattractant protein-1 (MCP-1) and macrophage inflammatory protein-1α (MIP-1α), through inhibition of the Notch signaling pathway, thereby alleviating colitis symptoms (105). CagA-containing exosomes from human gastric epithelial cells transcriptionally modulate the expression of claudin-2 via a caudal-type homeobox 2 (CDX2)-dependent mechanism, impairing intestinal mucosal repair. Additionally, exosomes from *H. pylori*-infected cells may compromise endothelial function, potentially increasing susceptibility to cardiovascular diseases (106). Macrophages exposed to exosomes rich in microRNAs from *H. pylori*-infected cells can either suppress *H. pylori* proliferation or induce pro-inflammatory responses, depending on the specific microRNA content. *H. pylori* also stimulates gastric epithelial cells to release circulating miR-25-enriched exosomes that modulate the NF-κB pathway via Kruppel-like factor 2 (KLF2). This modulation enhances the expression of intercellular adhesion molecule-1 (ICAM-1), MCP-1, vascular cell adhesion molecule 1 (VCAM-1), and IL-6, potentially accelerating the progression of atherosclerosis (107). Moreover, CagA within exosomes from *H. pylori*-infected gastric epithelial cells inhibits cholesterol transporter protein transcription by downregulating transcription factors such as peroxisome proliferator-activated receptor gamma (PPARγ) and liver X receptor alpha (LXRα), which promotes the formation of foam cells from macrophages (108). Additionally, CagA-rich exosomes from *H. pylori*-infected vascular endothelial cells induce reactive oxygen species generation in endothelial cells (109) (**Figure 5**).

## 7 Diagnostic and prognostic significance of OMVs and exosomes in *H. pylori*-associated diseases

Recent research emphasizes the significant role of OMVs and exosomes in managing *H. pylori* infections, especially concerning rising antibiotic resistance. These vesicles are increasingly recognized for their potential in immune modulation and targeted delivery of antimicrobial agents and as biomarkers in diagnostics. **Table 3** outlines key strategies that utilize OMVs and exosomes to enhance treatment efficacy and facilitate non-invasive detection methods for *H. pylori*-related diseases.

**Table 3:** Diagnostic and therapeutic applications of *H. pylori* OMVs and exosomes.

Category	Key Points	References
OMVs and Exosome-Based Strategies for targeted delivery of antimicrobial agents and immunomodulators	*H. pylori* OMVs and exosomes are promising candidates for immune modulation and targeted drug delivery in treating *H. pylori* infections, enhancing treatment efficacy while minimizing off-target effects.	(4, 21, 40, 110–112)
Engineering OMVs and Exosomes for specific targeting of *H. pylori* and modulation of host responses	OMVs and engineered exosomes have promising potential as innovative strategies for immune modulation and targeted therapeutic delivery in combating *H. pylori* infections, particularly in light of rising antibiotic resistance.	(3, 4, 30, 113–119)
Potential applications of OMVs and exosome therapy as an alternative or adjunct to antibiotics for *H. pylori*-related diseases	OMVs and engineered exosomes have promising potential as innovative strategies for immune modulation and targeted therapeutic delivery in combating *H. pylori* infections	(3, 4, 120, 121)
Identification of OMVs and exosomal biomarkers for early detection and monitoring of *H. pylori* infection	OMVs and exosomal biomarkers show significant potential in vaccine development and early detection of infections, facilitating robust immune responses and offering novel non-invasive diagnostic methods for effective management of *H. pylori*-related diseases.	(4, 9, 22, 39, 66, 122–124)
Non-invasive diagnostic tools utilizing *H. pylori* OMVs and exosomal biomarkers in the clinical management of *H. pylori*-associated diseases.	Recent studies highlight the potential of recombinant *H. pylori* OMVs and exosomal biomarkers as innovative diagnostic tools, enabling high-sensitivity tests and minimally invasive methods for early detection and monitoring of *H. pylori* infection and related diseases, thus enhancing personalized medicine approaches.	(125–131)

## 8 Challenges and future directions in investigating OMVs and exosomes associated with *H. pylori* infection

Understanding the pathogenicity of *H. pylori*, mainly through its OMVs and related exosomes, is essential for developing effective diagnostic and therapeutic strategies. However, current research in this area faces several limitations that impede a comprehensive understanding of the mechanisms of action and their translational applications. To address these challenges, we summarize the primary limitations of current research in **Table 4**. These limitations include challenges related to the standardization of isolation methods, the suitability of *in vitro* models, and gaps in our knowledge about the biogenesis and functions of exosomes in the context of *H. pylori* infection.

**Table 4:** Limitations of current research on OMVs and exosomes related to *H. pylori* infection.

Limitation	Description	References
Standardization of isolation methods	Current protocols for isolating OMVs and exosomes are not standardized, impacting reproducibility and clinical translation.	(18, 132–134)
*In vitro* model limitations	Most studies rely on *in vitro* models that do not accurately replicate the human gastrointestinal microenvironment, raising concerns about translational relevance.	(119, 135)
Understanding of biogenesis and release mechanisms	There is a lack of comprehensive understanding of how exosomes are produced and released from *H. pylori*-infected cells, including their specific cargo and functions.	(4, 135)
Genetic diversity in *H. pylori*	Significant genetic diversity among *H. pylori* strains affects OMV and exosome production, leading to inconsistent findings regarding their immunomodulatory effects.	(21, 117, 136)
Shape transitions of the bacterium	The shape transitions of the bacterium from spiral to curved, doughnut-shaped, and finally coccoid morphologies	(17, 19)
Specific signaling pathways and receptor interactions	The signaling pathways and receptor interactions involved in the immunomodulatory effects of OMVs and exosomes remain poorly characterized.	(37, 67, 68, 116)

Future research must adopt a focused approach to address these challenges effectively. **Table 5** presents key future research directions to improve our understanding of *H. pylori* OMVs and the effects of exosomes released by infected cells. These research directions emphasize the need to investigate the mechanisms of chronic infection, the interactions between exosomes and gastric microbiota, and the use of multi-omics techniques to clarify the complex interplay between *H. pylori* and the host immune response.

**Table 5:** Future research directions for *H. pylori* OMVs and infected cell exosomes.

Research Direction	Focus Area
Mechanisms of chronic infection	Investigate how *H. pylori* OMVs contribute to the mechanisms underlying chronic infection.
Cross-talk with gastric microbiota	Explore potential interactions between exosomes from *H. pylori*-infected cells and the gastric microbiota to understand disease progression and therapeutic outcomes better.
Multi-omics approaches	Utilize proteomics and metabolomics to analyze interactions between *H. pylori* OMVs and infected cell exosomes for a more comprehensive understanding of host-bacteria communication.
Exosome-centered therapies and diagnostics	Develop novel therapeutic and diagnostic approaches based on the properties of exosomes derived from *H. pylori*-infected cells.

## 9 Conclusion

In conclusion, this review focuses on the underexplored role of *H. pylori*-derived OMVs and exosomes in host immune modulation, aiming to provide new insights into the interactions between bacteria and host cells, with particular emphasis on the dynamic interplay mediated by these vesicles. Although current research has yielded valuable insights, challenges remain in fully elucidating the mechanisms underlying *H. pylori* OMVs and exosomes derived from infected cells. Future studies should prioritize integrating advanced techniques, such as single-cell sequencing and proteomics, and explore specific signaling pathways and molecular mechanisms to enhance our understanding of OMVs and exosomes. This approach will facilitate the development of innovative strategies for targeted therapeutic interventions in *H. pylori*-related diseases.
